# *Trichinella pseudospiralis vs. T. spiralis* thymidylate synthase gene structure and *T. pseudospiralis* thymidylate synthase retrogene sequence

**DOI:** 10.1186/1756-3305-7-175

**Published:** 2014-04-09

**Authors:** Elżbieta Jagielska, Andrzej Płucienniczak, Magdalena Dąbrowska, Anna Dowierciał, Wojciech Rode

**Affiliations:** 1 Nencki Institute of Experimental Biology, Polish Academy of Sciences, 3 Pasteur Street, 02-093 Warszawa, Poland; 2 Institute of Biotechnology and Antibiotics, 5 Starościńska Street, 02-516 Warszawa, Poland

**Keywords:** *Trichinella spiralis*, *Trichinella pseudospiralis*, Thymidylate synthase, Gene structure, Introns-late theory, Retrogene

## Abstract

**Background:**

Thymidylate synthase is a housekeeping gene, designated ancient due to its role in DNA synthesis and ubiquitous phyletic distribution. The genomic sequences were characterized coding for thymidylate synthase in two species of the genus *Trichinella*, an encapsulating *T. spiralis* and a non-encapsulating *T. pseudospiralis*.

**Methods:**

Based on the sequence of parasitic nematode *Trichinella spiralis* thymidylate synthase cDNA, PCR techniques were employed.

**Results:**

Each of the respective gene structures encompassed 6 exons and 5 introns located in conserved sites. Comparison with the corresponding gene structures of other eukaryotic species revealed lack of common introns that would be shared among selected fungi, nematodes, mammals and plants. The two deduced amino acid sequences were 96% identical. In addition to the thymidylate synthase gene, the intron-less retrocopy, i.e. a processed pseudogene, with sequence identical to the *T. spiralis* gene coding region, was found to be present within the *T. pseudospiralis* genome. This pseudogene, instead of the gene, was confirmed by RT-PCR to be expressed in the parasite muscle larvae.

**Conclusions:**

Intron load, as well as distribution of exon and intron phases in thymidylate synthase genes from various sources, point against the theory of gene assembly by the primordial exon shuffling and support the theory of evolutionary late intron insertion into spliceosomal genes. Thymidylate synthase pseudogene expressed in *T. pseudospiralis* muscle larvae is designated a retrogene.

## Background

*Trichinella spiralis* and *Trichinella pseudospiralis* are two parasitic nematode species colonizing mammalian striated muscles. Transmission of *Trichinella* spp. to the next host occurs by ingestion of meat contaminated with the parasite muscle larvae. In the intestine, after mating, adult female worms give birth to the newborn larvae which migrate to the muscles to become the muscle larvae [1]. *T. spiralis* is an encapsulating species whose muscle larvae reside in discrete structures called nurse cells, separated from myofibers by collagen capsules [2,3]. Instead, muscle larvae of *T. pseudospiralis*, migrate freely throughout the muscle tissue [1]. Our previous studies documented high thymidylate synthase activity persisting in developmentally arrested muscle larvae of both species, as well as in *T. spiralis* adult forms [4,5]. Thymidylate synthase (EC 2.1.1.45), catalyzing deoxyuridylate methylation to yield a DNA precursor, thymidylate, is the only cellular source of *de novo* synthesis of the latter [6]. High enzyme activity is known to accompany proliferation, as well as to persist in certain growth-arrested systems where it is not associated with cell division cf. [5]. Consequently, while the enzyme expressed in embryos developing in the *T. spiralis* adult uterus may be considered a marker of proliferation, its localization to excretory-secretory organ, i.e. stichosome, of *T. spiralis* adult forms, as well as to gonads and stichosome primordial cells of non-growing muscle larvae, appears to reflect the state of cell cycle arrest [7]. Of note is that high thymidylate synthase activity found in *T. spiralis* and *T. pseudospiralis* muscle larvae does not appear to vary in connection with the difference in the intracellular niche occupied by two species.

Thymidylate synthase belongs to the proteins most highly conserved in evolution [6]. It is encoded by a housekeeping gene, considered ancient by virtue of the enzyme’s role in DNA synthesis and its ubiquitous phylogenetic distribution [8]. This implies that the gene was likely to play a role in the transition from an RNA to a DNA world and to exist in the common ancestor of modern organisms before kingdoms diverged [9]. Analyses of evolutionary pathways of thymidylate synthase and other ancient genes may serve delineation of the origin of modern DNA sequences and the molecular basis of their evolutionary dynamics [10]. Those genes are also used as models for studying spliceosomal intron distribution among phylogenetic lineages, as the issue of intron loss or gain during evolution of eukaryotic protein-coding genes remains unsettled [11]. Since the discovery of introns, two concepts of their origin have been considered: (i) the introns-early theory or exon theory of genes assumes gene assembly by primordial exon shuffling in a common ancestor of bacteria and eukaryotes, and subsequent massive intron loss over evolution, and (ii) the introns-late theory or insertional theory assumes considerable intron gain during recent times [12]. Of note is that the two theories are also viewed as unlinked rather than incompatible, since introns present in the primordial genes might have been removed and new ones rapidly inserted in different positions [13]. It is also commonly agreed that the last eukaryotic common ancestor had a high intron density [14,15].

*T. spiralis* thymidylate synthase cDNA has been cloned, allowing determination of the nucleotide and deduced amino acid sequences [16] that turned out to be identical with the gene coding sequence inferred from the recently published draft version of the *T. spiralis* genome [17]. Phylogenetic analysis of amino acid sequences corresponding to the enzymes of different specific origin showed *T. spiralis* thymidylate synthase to branch off before other nematode species and display higher overall similarity with mammalian than other nematode enzymes [16]. Early divergence of the genus *Trichinella* in the evolution of the phylum Nematoda, as well as differences in the genome characteristics of *Trichinella* in relation to other nematode lineages were also documented by others [18,19].

The goal of the present study was to characterize the *T. pseudospiralis* thymidylate synthase cDNA and genomic sequences, in order to perform analysis of introns distribution and compare it with that of the corresponding *T. spiralis* gene determined previously [20]. Considering previously suggested evolutionary distinctness of the genus *Trichinella* in the evolution of the phylum Nematoda (see above), of interest was also to compare both *Trichinella* thymidylate synthase genes with the corresponding genes of various eukaryotic species.

## Methods

### Primer sequences

IVTF: 5′ GGGTCTAGACTTGAATGTTATAGATATTTATACAATG 3′, IVTR: 5′ GGGAAGCTTCCATGAAATTTTATTTC 3′, RGENP: 5′ GAGAGCGGCCGCCAATGACAGAAACTGTTCACAAATTAG 3′, RGENK: 5′ AAAGCGGCCGCGATCACACAGCCATAGGCATTG 3′, RGEN5′1: 5′ AAAAGCGGCCGCACGTAATCATCCTGAGATG 3′, RGEN5′2: 5′ AAAAGCGGCCGCAGCTTTCAGAGAAGAATGTC 3′, RGEN3′1: 5′ GAGAGCGGCCGCCTATGGCTGTGTGATCAATTG 3′, RGEN3′2: 5′ GAGAGCGGCCGCCCAAATCACCTTCTTCATAATTG, SYNEX1: 5′ GGGGATCCATATGACAGAAACTGTTCACAAATTAG 3′, SYNEX2: 5′ AAAAGCTTACACAGCCATAGGCATTGATA 3′.

### Biological material

*T. spiralis* (ISS 569 155 569) and *T. pseudospiralis* muscle larvae were maintained and isolated, as previously described [5].

### Nucleic acids isolation

Total RNA was isolated from *T. pseudospiralis* muscle larvae using TRIZOL reagent (Life Technologies) and genomic DNA from muscle larvae of both species was extracted using Wizard Genomic DNA Purification Kit (Promega), with the manufacturer’s protocols applied in each case.

### Reverse transcription

This was performed on total RNA prepared from *T. pseudospiralis* muscle larvae with SYNEX2 primer, in two rounds of 1 h incubation at 42°C [16], using MMLV reverse transcriptase (Promega).

### Polymerase Chain Reaction (PCR)

In order to amplify *T. pseudospiralis* thymidylate synthase ds cDNA, as well as *T. spiralis* and *T. pseudospiralis* thymidylate synthase genes, standard PCR on ss cDNA or genomic DNA was performed, using *Taq* DNA polymerase (Promega), as recommended by the enzyme manufacturer. The following primer combinations and cycling conditions were applied: (i) in the case of *T. pseudospiralis* thymidylate synthase cDNA, SYNEX1 and SYNEX2 primers were used, with initial 3 min at 95°C and the hot start steps, followed by 35 cycles of 30 sec at 95°C, 30 sec at 57°C and 1 min at 72°C, (ii) in the case of *T. spiralis* gene, RGENP and RGENK primers were used, with initial 2 min at 95°C and the hot start steps, followed by 3 cycles of 15 sec at 95°C, 15 sec at 60°C and 2 min at 72°C, and subsequent 29 cycles of 15 sec at 95°C, 1 min at 68°C and 2 min at 72°C, (iii) in the case of *T. pseudospiralis* gene, IVTF and IVTR primers were used, with cycling conditions as in (ii), but for the annealing step being performed at 40°C in the initial 3 cycles and at 58°C in the subsequent 29 cycles. For each amplification a negative control was included. Amplification products were analyzed by electrophoresis in Tris-acetate-EDTA buffered 0.8% agarose gels.

### Inverse PCR

In order to determine *T. spiralis* thymidylate synthase gene 5′ and 3′ flanking regions, inverse PCR was used [21]. Genomic DNA extracted from *T. spiralis* muscle larvae was digested for 1 h at 37°C with *Eco* RI (Life Technologies), then ligated overnight at 16°C using phage T4 DNA ligase (Promega). A 1/100 dilution of the ligation mixture served as a template for PCR with *Taq* DNA polymerase (Promega), applied as recommended by the enzyme manufacturer. In the case of the 5′ region amplification, RGEN5′1/RGEN5′2 primers and the following cycling steps were applied: (i) 2 min at 95°C, followed by the hot start step, (ii) 3 cycles of 15 sec at 95°C, 15 sec at 50°C and 2 min at 72°C, (iii) 29 cycles of 15 sec at 95°C, 1 min at 68°C and 2 min at 72°C. In the case of the 3′ region amplification, RGEN3′1/RGEN3′2 primers were used and cycling conditions as described above, but for the annealing step during the initial 3 cycles performed at 60.5°C. For each amplification a negative control was included. Amplification products were analyzed by electrophoresis in Tris-acetate-EDTA buffered 0.8% agarose gels.

### Cloning and sequencing

cDNA corresponding to *T. pseudospiralis* thymidylate synthase coding sequence was cloned into *Bam* HI and *Hind* III sites of pBluescript SK (+) phagemid (Agilent Technologies) propagated in *E. coli* DH5αF’ strain. DNA amplicons corresponding to *T. spiralis* thymidylate synthase gene and gene flanking regions were cloned into *Not* I site of the same vector. DNA corresponding to *T. pseudospiralis* thymidylate synthase gene was cloned into *Xba* I and *Hind* III sites also into pBluescript SK (+) vector. Sequencing of cloned *T. spiralis* genomic DNA fragments was performed using Sequenase Version 2.0 DNA Sequencing kit (Affymetrix) and sequencing of *T. pseudospiralis* thymidylate synthase cDNA and genomic DNA was done by DNA Sequencing and Oligonucleotide Synthesis core facility at the Institute of Biochemistry and Biophysics (Warsaw, Poland).

### GenBank submissions

*T. spiralis* and *T. pseudospiralis* thymidylate synthase gene sequences were deposited in the GenBank and are available under accessions [GenBank:AF406808] and [GenBank:KF186231], respectively.

### Protein secondary structure analysis

Crystal structures of thymidylate synthase protein monomers complexed with enzyme substrate deoxyuridylate, dUMP, were obtained from Protein Data Bank ( http://www.rcsb.org), under the accession codes [PDB:4G9U] for *T. spiralis*, [PDB:4E5O] for *M. musculus* and [PDB:3HB8] for *H. sapiens* R163K mutated enzyme. Secondary structure element assignment for subunit A of each model, was determined by PDB managing system. Graphical structure representation was obtained using VMD 1.8.7 software, implemented with “vmd_use_pdb_ss” script enabling reading of secondary structure elements from PDB files at http://www.ks.uiuc.edu/Research/vmd/.

### Ethical approval

Ethical approval was granted by the First Warsaw Local Ethics Committee for Animal Experimentation at the Nencki Institute.

## Results

### *T. spiralis* and *T. pseudospiralis* thymidylate synthase genes share the same structure and show 11 substitutions at the deduced amino acid sequence level

**Figure 1:**
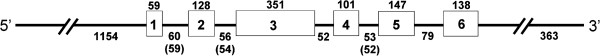
**Structures of genes of *T. spiralis *and *T. pseudospiralis *thymidylate synthases.** Exons and introns are shown as boxes and lines, respectively. The lengths of *T. spiralis* exons, as well as gene flanking and intronic regions, are given above and below the scheme, respectively. *T. pseudospiralis* intron lengths when different from *T. spiralis*, are given in parentheses. The alignment of two sequences is shown as Additional file 1: Figure S1.

*T. spiralis* and *T. pseudospiralis* thymidylate synthase genes consist of 6 exons, intervened by 5 introns, marked by GT/AG donor/acceptor splice sites (Figure 1). All exons are of the same length and 54 single nucleotide substitutions in 52 codons are found between two species within the 924 nt-long open reading frame (Additional file 1: Figure S1). Out of those, 37 substitutions are identified as transitions, i.e. changes between two purines or two pyrimidines, and 17 are identified as transversions, i.e. changes of purine into pyrimidine and vice versa. Transitions, being twice as frequent as transversions, reminded of the pattern of nucleotide substitutions inferred for human genome based on its pseudogene sequences analysis [22]. Among 54 nucleotide substitutions 42 are silent and 12 result in changes of deduced protein sequence. *T. spiralis* and *T. pseudospiralis* thymidylate synthase amino acid sequences show 96% identity (Additional file 1: Figure S2). The entire 2794 nt-long *T. spiralis* thymidylate synthase gene sequence [GenBank:AF406808] shows 99% overall identity (BLAST comparison), with the sequence of the corresponding region of *T. spiralis* genome draft [GenBank:NW_003526941]. In both versions the sequences of exons, introns and gene 3′ flanking regions are identical. Several single nucleotide differences, i.e. 10 insertions, 1 substitution and 2 deletions, were found only within gene 5′ flanking regions. Nine of those differences appear at the sites of single nucleotide stretches, thus resulting presumably from sequence reading obstacles. Within 340 nt of the gene 5′ flanking region consensus TATA box was identified (Additional file 1: Figure S1), implying transcriptional regulation of the parasite gene to be different from mammalian, whose promoters are TATA-less [23].

### Two conserved introns and one homologous are present in *Trichinella* and mammalian thymidylate synthase genes

**Figure 2:**
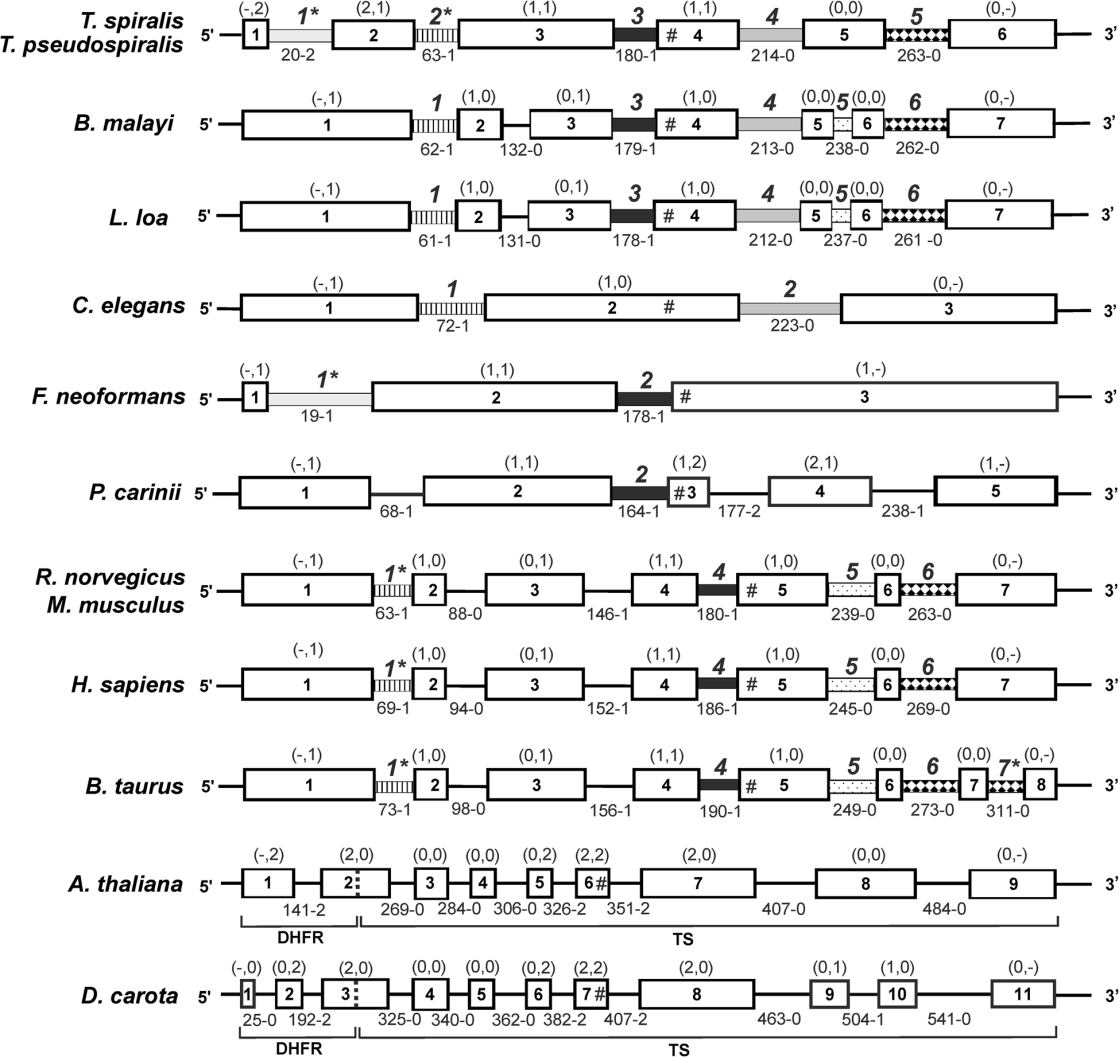
**Comparison of gene structures of various thymidylate synthases.** Exons are boxed and introns are shown as lines. The introns are annotated below the gene schemes with the number of codons they break within particular genes and with their phase, being 0 when falling between codons, and 1 or 2 when interrupting codons. Introns located in conserved or homologous positions, are marked with the same line pattern and the intron number within particular species gene. The positions of slide introns (5-nt slide in the case of *Trichinella* intron 1 vs. *F. neoformans* intron 1, and 3-nt slides in all other cases), are marked with asterisks. Exons are annotated with their numbers within particular genes and their phases given in the brackets. Number key within particular exon box indicates location of the enzyme active center. Dihydrofolate reductase (DHFR) and thymidylate synthase (TS) parts of bifunctional plant genes are marked below the gene schemes. Gene sequences that served for exon-intron junctions determination were obtained through the following accessions: *Brugia malayi* [GenBank:NW_001893010], *Loa loa* [GenBank:NW_003320690], *Caenorhabditis elegans* [GenBank:AF099673.1], *Filobasidiella neoformans* [GenBank:U12256.1], *Pneumocystis carinii* [GenBank:M25415.1], *Rattus norvegicus* [GenBank:NC_005108.3], *Mus musculus* [GenBank:NW_001030787.1], *Homo sapiens* GenBank:NC_000018.9], *Bos Taurus* [GenBank:GJ062838.1], *Arabidopsis thaliana* [GenBank:NC_003071.7], *Daucus carota* [GenBank:AJ003139.1]. Amino acid sequence alignment with marked intron positions is shown as Additional file 1: Figure S3.

Intron distribution within *T. spiralis/T. pseudospiralis* thymidylate synthase genes was compared with gene structures of other species (Figure 2). The genes of the free-living nematode *C. elegans* and fungus, *F. neoformans*, the latter being an airways pathogen of immunocompromised patients, contain low intron load, i.e. 2, vs. 4 present in other airways pathogenic fungi species, *P. carinii*, and 5 present in the *Trichinella* gene. The genes of two other parasitic nematodes, *B. malayi* and *L. loa*, contain 6 introns, as is also the case for mammalian genes, including human (only *B. taurus* carries one additional intron). Plant genes appear the richest in introns, with *D. carota* carrying 8 introns within the thymidylate synthase part of its bifunctional dihydrofolate reductase/thymidylate synthase gene. Conserved introns, i.e. present in exactly the same positions, or homologous introns, i.e. those whose positions are shifted (slid) by several nucleotides, are found among animal and fungal genes. Among nematode thymidylate synthase genes four conserved introns are present in *Trichinella*, *B. malayi* and *L. loa* genes. Out of those four, two introns corresponding to *Trichinella* introns 2 and 4, are also found in *C. elegans* gene, as its only intron burden. Within mammalian thymidylate synthase genes all introns are conserved, with the exception of *B. taurus* intron 7 which remains shifted by 3 nt in relation to the terminus of the corresponding intron 6 present in other mammalian species. The genes of nematodes, with the exception of *C. elegans*, and those of fungi and mammals contain one conserved intron, corresponding to *Trichinella* intron 3. This most highly conserved intron is located 11 nt upstream from the junction site of the exon carrying the region coding the enzyme active center. Both plant, *A. thaliana* and *D. carota* genes contain an intron located 12 nt downstream, and *P. carinii* carries an additional intron located 8 nt downstream from that region (intron positions marked on the aligned amino acid sequences are provided as Additional file 1: Figure S3). Thus *P. carinii* enzyme active center is encoded by a separate, 40 nt-long exon. The distance between animal/fungal and plant introns situated on the opposite sites of this region, is 44 nt. Apart from *Trichinella* intron 3, animal (with the exception of *C. elegans*) thymidylate synthase genes contain an additional conserved intron, corresponding to *Trichinella* intron 5. Additionally, a homologous intron, corresponding to *Trichinella* intron 2, is present in animal genes, with 3 nt-shift in mammalian vs. nematode genes. Besides, a conserved intron, absent from *Trichinella* and *C. elegans* genes, is present in *B. malayi*, *L. loa* and mammalian genes as intron 5. In regard to other intron position homology, *F. neoformans* and *Trichinella* genes carry intron 1 shifted by 5 nt between two genes. Introns in plant *A. thaliana* and *D. carota* thymidylate synthase, parts of the bifunctional genes, are found in the conserved positions with the exception of an additional *D. carota* intron 7. Yet no common intron is found for plant vs. animal and fungal genes. Intron positions may breake coding sequence between codons (intron phase 0), or after the first or the second base pair (intron phase 1 or 2, respectively) [24]. In fungal and animal thymidylate synthase genes, with the exception of those of *B. malayi*, *L. loa* and *B. taurus*, the intron phases other than 0 are dominating or equally frequent. In *B. malayi*/*L. loa*, *B. taurus*, *A. thaliana* and *D. carota* genes, phase 0 introns prevail, reaching the numbers 4 out of 6, 4 out of 7, 5 out of 7 and 5 out of 8, respectively. In analogy to the intron phases, also exon phases are defined. The exons beginning and ending with the same phase are called symmetric, in contrast to asymmetric ones, which begin and end with a different phase [24]. In *Trichinella* and *A. thaliana* thymidylate synthase genes symmetric exons prevail, reaching the numbers 4 out of 6 and 5 out of 8, respectively. In *P. carinii*, gene asymmetric exons significantly prevail (4 out of 5). In *B. malayi*, *L. loa* and mammalian genes, asymmetric exons are more frequent (4 out of 7). This is also the case for *C. elegans* (2 out of 3), *F. neoformans* (2 out of 3) and *D. carota* (5 out of 9), genes. In *B. taurus* the frequency of both types of exons is equal.

### Lack of correlation among location of conserved and homologous introns and the protein secondary structure entities

**Figure 3:**
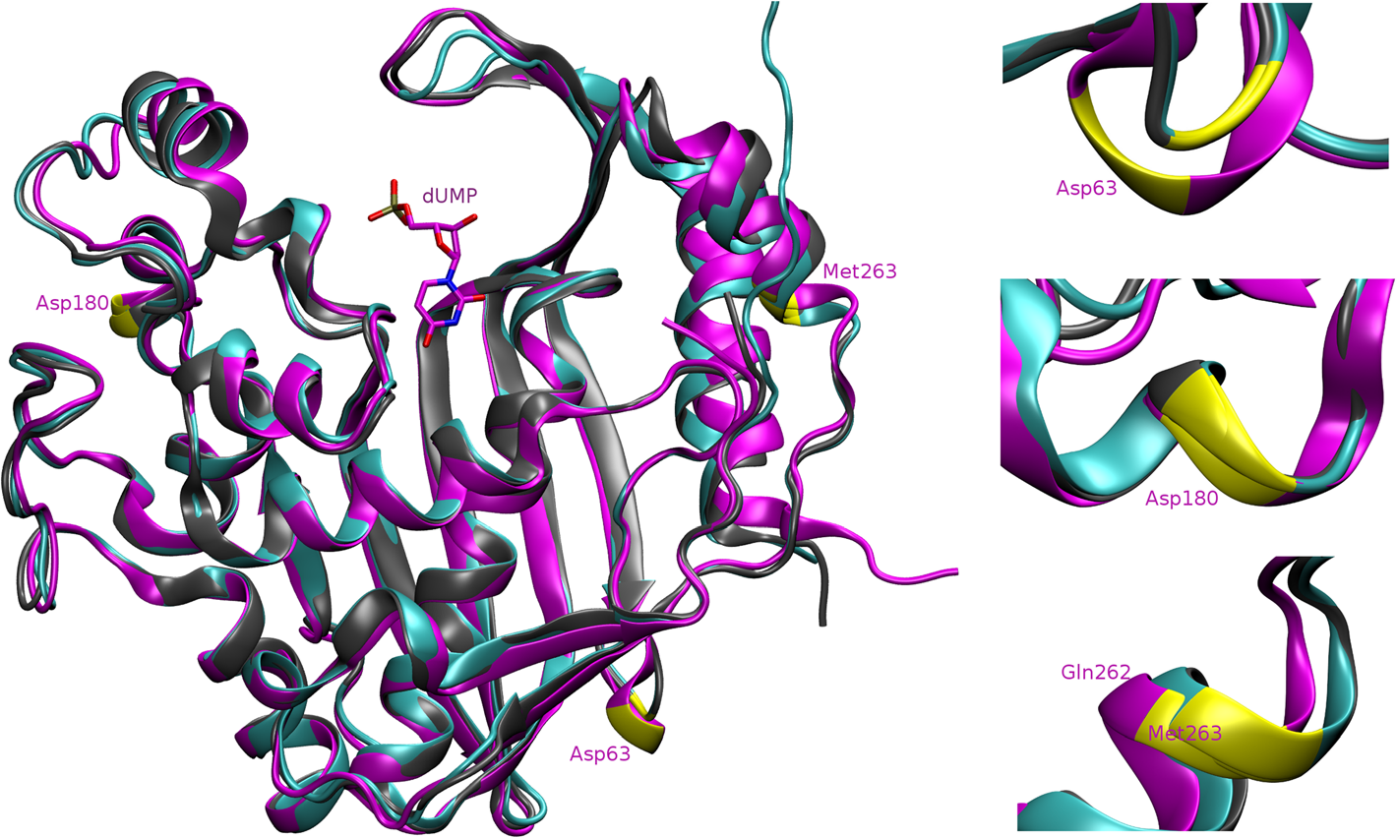
**Superimposition of *T. spiralis *(magenta), mouse (cyan) and human (grey) thymidylate synthase structure models of subunits A of dUMP complexes, obtained through the accessions [PDB:4G9U], [PDB:4E5O], [PDB:3HB8], respectively.** Secondary structure elements are distinct with helices shown as ribbons, β-sheets as wide parallel arrows and unclassified regions shown as lines. Derivative sites of homologous intron located at Asp-63 and two conserved introns located at Asp-180 and Gln-262/Met-263 (each amino acid and its number is given for *T. spiralis* sequence), are marked in yellow with corresponding structural elements shown aside in enlargement.

Location within amino acid sequence of splicing sites of three introns, two conserved and one homologous among *T. spiralis*, *M. musculus* and *H. sapiens* thymidylate synthase genes, and assignment of those sites to the protein spatial structure in enzyme models obtained from PDB, were followed (Figure 3). *T. spiralis* intron 2 and homologous mammalian intron 1, both being phase 1 introns (Figure 2), do not separate secondary structure elements. The former lays within a helix and the latter is located within a region assigned in PDB files neither helical nor β-sheet form. While *Trichinella* intron 3 is located also within a helix, conserved with it mammalian intron 4 is located at the edge of a helix, despite both introns being of phase 1. *Trichinella* intron 5 and conserved mammalian intron 6, both being phase 0 introns, are located at the edge of helices. No correlation can thus be inferred among intron locations, their phases, and protein secondary structure element borders.

### Thymidylate synthase retrogene is expressed instead of the gene in *T. pseudospiralis* muscle larvae

**Figure 4:**
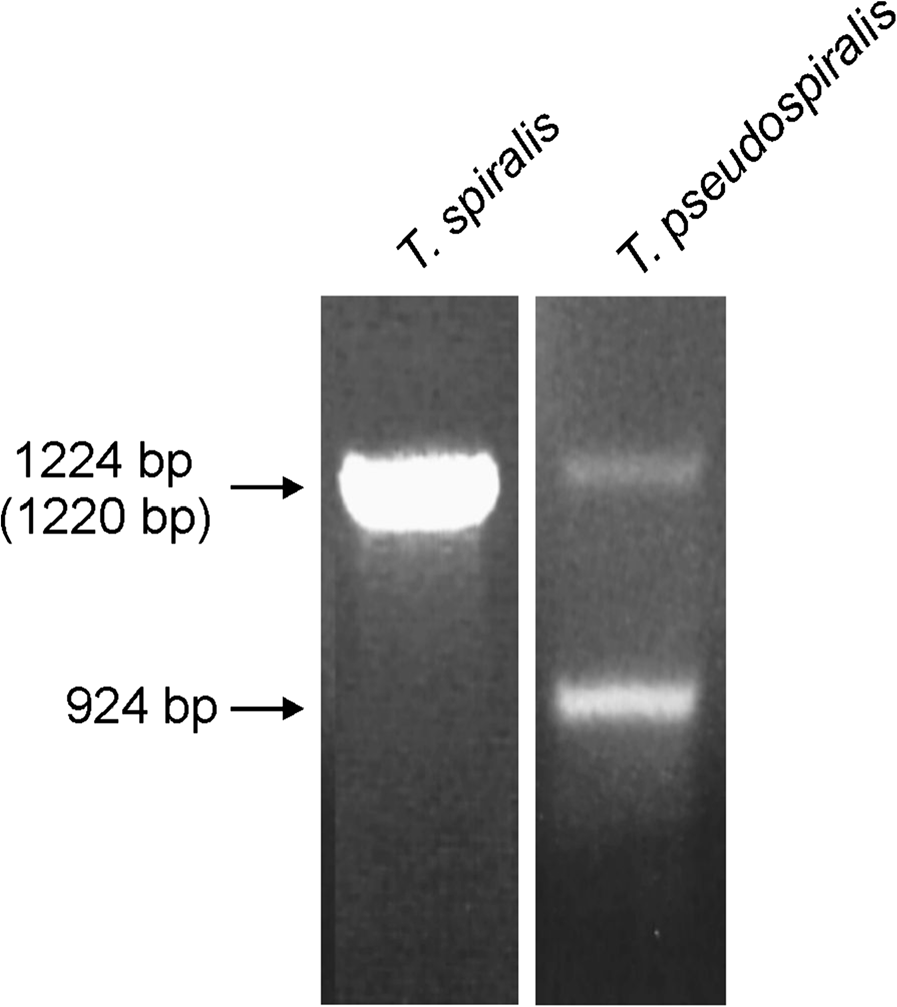
**Electrophoretograms of PCR products resulting from amplification of *T. spiralis *and *T. pseudospiralis *genomic DNA, performed with primers specific for the ends of *T. spiralis *thymidylate synthase ORF.** The accurate lengths, based on sequencing, are given for *T. spiralis* and *T. pseudospiralis* (in parentheses) genes, and for *T. pseudospiralis* retrogene.

PCR on *T. pseudospiralis* genomic DNA resulted in two products, a 1220 nt-long amplicon, corresponding to the gene region encompassing exon and intron sequences, and a 924 nt-long amplicon of the sequence identical to *T. spiralis* thymidylate synthase ORF, designated a pseudogene (Figure 4). RT-PCR on RNA isolated from *T. pseudospiralis* muscle larvae used as a starting material resulted in a single product (not shown), and sequencing of several bacterial clones revealed its sequence to be identical with that of *T. spiralis* thymidylate synthase cDNA. In view of the latter, a processed pseudogene is apparently expressed in *T. pseudospiralis* muscle larvae. Ultimately, its genomic sequence is referred to as retrogene. Although none of the clones sequenced carried a sequence that would correspond to *T. pseudospiralis* thymidylate synthase gene, transcription of the gene cannot be unequivocally excluded.

## Discussion

In the present study *T. spiralis* and *T. pseudospiralis* thymidylate synthase genes were found to share exon-intron structure. Comparison with the gene structures of other eukaryotes, including animal, fungal and plant species, revealed lack of a common intron. However, a conserved intron is found in the vicinity of the region encoding the enzyme active center in nematode (with the exception of *C. elegans*), fungal and mammalian genes. In *T. pseudospiralis* genomic DNA, apart from the gene, thymidylate synthase retrogene was identified, of the sequence identical to *T. spiralis* thymidylate synthase ORF. This retrogene was found to be expressed, instead of the gene, in the parasite muscle larvae.

Comparison of intron distribution among *Trichinella* and other eukaryotic thymidylate synthase genes shows lack of a conserved or even slid intron that would be shared by the organisms included in the analysis, representing animal, plant and fungal crown groups. The intron sliding, termed also intron slippage or drifting, indicates either preexisting intron relocation to a nearby position or its replacement by a new intron at a nearby position. This phenomenon is proposed to be associated with alternative splicing, proceeding by reverse splicing mechanism, i.e. insertion of a spliced-out intron into gene transcript, followed by reverse transcription and homologous recombination. Intron slippage is considered to reflect intron gain events rather than indicate location of an ancient intron, i.e. being remnant from the eukaryotic common ancestor [11,15,25]. Among the species studied, the most highly conserved is the intron corresponding to *Trichinella* intron 3, present also in other nematodes (with the exception of *C. elegans*), fungal and mammalian genes. As absent from plant genes, it cannot be considered ancient but rather constituting a relic from the common ancestor of fungi and animal evolutionary lineages [26]. Commonality of intron load within animal lineage only is further evident, based on the presence in all nematodes and mammals of an intron homologous to *Trichinella* intron 2 and conservation of two other introns, one corresponding to *Trichinella* intron 5 and present in all other species, and the other, absent from *Trichinella* and *C. elegans* genes but present in *B. malayi*, *L. loa* and mammalian genes as intron 5. An overall lower intron number in *C. elegans* than in other nematode thymidylate synthase genes (2 vs. 5/6), remains in agreement with a notion that *C. elegans* phylogeny, unlike with other nematodes, is characterized by extensive intron loss and restricted intron gain [14,27]. Thus excluding *C. elegans*, which does not seem to be a representative model for spliceosomal intron studies, it can be inferred that thymidylate synthase genes of all animal species included in the analysis represent a similar intron location pattern. Also plant thymidylate synthase genes show apparently kingdom-specific intron distribution. Therefore, it can be hypothesized that locations of introns in thymidylate synthase genes represent new insertional events, occurring independently in animal and plant kingdoms, with fungi sharing possibly a common thymidylate synthase gene origin with animals. The aforementioned conservation of *Trichinella* intron 3 in nematodes (with the exception of *C. elegans*), fungal and mammalian genes may be associated with the downstream proximity of the enzyme active center-coding region. Also in plant *A. thaliana* and *D. carota* genes, there is an intron located proximally, but downstream from that region highly conserved among various species [6]. The distance (44 nt) between locations of fungal/animal and plant introns, on the opposite sites of the active center coding-sequence, is too long to result from intron sliding (up to 15 nt are allowed for a slide), and rather too short for coding a functional peptide, considering an assumed minimum evolutionary exon length of 45 base pairs [26]. Therefore, it is not likely that both introns were present in the last common eukaryotic ancestor. Their locations seem to point rather to late insertional events, occurring in the vicinity of 21 base pairs-long region coding the enzyme active center, which remained uninterrupted due to selection pressure. Additionally, only in *Trichinella* and plant genes the active center-containing exon is symmetric, unlike in other nematode, fungal and mammalian genes, where it is asymmetric. Exon symmetry is claimed to be required for maintenance of the reading frame in the case of exon shuffling [24]. Thus in the majority of thymidylate synthase genes analyzed, the active center-coding exon does not conform to this criterion. According to the exon shuffling theory, not only symmetric exons but also phase 0 introns are believed to be favored [24]. Such intron positions are not predominant in *Trichinella*, *C. elegans*, fungal and mammalian genes, except in that of *B. Taurus*, but in *B. malayi*, *L. leo*, *B. taurus* and plant genes phase 0 introns prevail. However, a high number of phase 0 introns occurs in intron-rich regions and appears correlated with higher overall intron load. Also, in animal and plant thymidylate synthase genes, short exons, associated with phase 0 intron-rich regions, tend to be symmetric. Thus, based on the distribution of intron and exon phases within thymidylate synthase genes, and in light of the lack of an ancient intron, the rules apparently governing the evolutionary exon shuffling seem to apply to introns late insertional events. A positive correlation between high intron density and species developmental complexity has been established [13] and an overall high intron complement, evident for mammalian and plant thymidylate synthase genes, conforms to that rule. However, the intron load, similarly high in nematodes, with the exception of *C. elegans*, and mammalian genes, casts doubt on this statement. Interestingly, while the two *Trichinella* species display thymidylate synthase gene structure similar to other nematodes (with the exception of *C. elegans*), the enzyme sequence similarity analysis implicates early evolutionary divergence of the genus *Trichinella* from the phylum Nematoda. This observation indicates that sequence and gene structure evolution may not be closely linked phenomena. In summary, analysis of gene structures of various thymidylate synthase genes provides support for the introns-late theory, pointing to a recent acquisition of the introns in the course of eukaryotic spliceosomal gene evolution. This conclusion is also supported by an apparent lack of correlation between conserved or homologous intron locations and the positions of the edge amino acid residues of thymidylate synthase protein secondary structure entities. In respect to the latter, intron insertion seems to proceed rather as a stochastic event. Analogous conclusions were inferred also from the intron load pattern in other ancient housekeeping genes, including actin [28], glyceraldehyde-3-phosphate dehydrogenase [29], triose-phosphate isomerase [12,26,30] and tubulin [31].

The present paper reports on thymidylate synthase retrogene identified in *T. pseudospiralis* muscle larvae. Pseudogenes are defective copies of functional genes that accumulated in the genomes of many modern organisms, especially mammals. They tended to be considered as molecular relics, accumulating numerous mutations due to release from selection pressure [32]. Pseudogenes may arise either by duplication of genomic DNA or by retroposition, i.e. reverse transcription primed at poly A tails of an intron-less transcript, followed by insertion of a transposable element into another genomic location. Pseudogenes arising by the second mechanism, called processed pseudogenes or retropseudogenes, display a low survival rate [33,34]. Only 10% of protein coding genes of the human genome have been estimated to have at least one processed pseudogene. Processed pseudogenes of highly expressed housekeeping genes, including those coding for ribosomal proteins, keratin, glyceraldehyde-3-phosphate dehydrogenase and actin, account for the majority of the total number of ~8000 human processed pseudogenes [35]. Mouse genome was estimated to contain ~5000 processed pseudogenes [36]. Thymidylate synthase processed pseudogene incapable of encoding functional enzyme was reported for a mouse fibroblast cell line [37]. Among human and mouse genes, those having multiple copies of processed pseudogenes are predominantly housekeeping genes highly expressed in the germ lines or embryonic cells [36]. Although the vast majority of pseudogenes remain functionally inactive, the evidence is accumulating on the abundance of functional processed pseudogenes, called retrogenes, especially in mammals and insects [33]. In particular, 20% of human genome processed pseudogenes are believed to be expressed [34].

To our best knowledge, it is not only the first retrogene of a housekeeping gene, but also the first pseudogene in general, described for genus *Trichinella. C. elegans* genome was estimated to contain ~2000 pseudogenes [38]. However, this number may not be meaningful for *Trichinella* whose thymidylate synthase protein sequence [16], as well as gene structure (vide supra), are apparently divergent from those of *C. elegans*. Understanding the mode of thymidylate synthase retrogene insertion into *T. pseudospiralis* genome and its transcriptional regulation require further investigation. However, as the muscle larvae of this species move freely within the muscle tissue, in contrast to *T. spiralis* muscle larvae being confined to a capsule, a possibility appears that the retrogene expression accounts for the life style adaptation (*cf*. [39]). In the absence of the capsule, overall transcription regulation may require hypertranscription of thymidylate synthase gene, in order to maintain high enzyme activity, and consequently high levels of its product. This type of regulation is known to take place for retrogenes of broadly expressed housekeeping genes, located to the autosomal chromosomes during and after male meiosis [33]. This reasoning corresponds with a notion that the energy demands of transcription and splicing may favor selection for compactness in the case of highly expressed and/or rapidly regulated genes [40,41], and thymidylate synthase was identified as highly expressed in *Trichinella* muscle larvae.

The mechanisms of both phenomena discussed in this paper in regard to *Trichinella* thymidylate synthase genomic sequences, i.e. relatively high intron load and retroposition, are underlain by reverse transcriptase activity. Thus, high levels of reverse transcription seems to shape genome evolution of this lineage. Analysis of other processed pseudogenes and their flanking sequences is required, in order to identify a putative general mode of retroposition specific for this genus. In mammals, involvement of the L1 LINE retrotransposon has been established [42,43].

## Conclusions

Intron load and distribution within thymidylate synthase genes of various species display kingdom-specific patterns with no conserved or homologous introns shared among fungi, animals and plant genes. Locations of conserved and homologous introns within animal genes do not show correlation with the enzyme protein structural motifs borders. This allows us to conclude that intron insertion into thymidylate synthase genes depicts evolutionary late gain events, being rather stochastic in regard to the enzyme structure. Identification of thymidylate synthase retrogene in *T. pseudospiralis* muscle larvae points to a possibility that compactness of genomic sequence coding for this enzyme may reflect larval adaptation to existence within the muscle tissue in a non-encapsulated form.

## Competing interests

The authors declare that they have no competing interests.

## Authors’ contribution

EJ performed PCR, molecular cloning and sequencing. AP designed the experiments of fragment amplification and cloning. MD designed data presentation and interpretation. AD performed protein structure studies. WR designed the project and coordinated its implementation. All authors read and approved the final version of the manuscript.

## Supplementary Material

Additional file 1: Figure S1 Alignment of *T. spiralis* and *T. pseudospiralis* thymidylate synthases exon (bold) and intron (italic) sequences. Additionally, a 98 nt-long fragment of the *T. spiralis* gene 5′ flanking region is shown, with putative TATA box underlined, and 29 nt-long fragment, corresponding to 3′ UTR [16], is shown as the gene 3′ flanking region. The whole *T. spiralis* and *T. pseudospiralis* gene sequences are available through the accessions [GenBank:AF406808] and [GenBank:KF186231], respectively. Nucleotide positions differing between the genes of two species are indicated by asterisks. Alignment was performed using Clustal X software and Genomatix MatInspector software served for consensus TATA box identification. **Figure S2.** Alignment of *T. spiralis* and *T. pseudospiralis* thymidylate synthase amino acid sequences. Amino acid substitutions are marked with asterisks. Enzyme conserved folate and doexyuridylate (active center) binding sites are marked bold. **Figure S3.** Alignment of amino acid sequences of various thymidylate synthases performed in Clustal X software. The intron positions are marked as boxes for phase 0 introns, elipses for phase 1 introns or pentagons for phase 2 introns. The sequences were obtained via the following accessions: *Trichinella spiralis* [GenBank:AF406808.3]*, Trichinella pseudospiralis* [GenBank:KF186231], *Brugia malayi* [GenBank:NW_001893010], *Loa loa* [Genbank:NW_003320690], *Caenorhabditis elegans* [GenBank:AF099673.1], *Filobasidiella neoformans* [GenBank:U12256.1], *Pneumocystis carinii* [Genbank:M25415.1], *Rattus norvegicus* [GenBank:NC_005108.3], *Mus musculus* [GenBank:NW_001030787.1], *Homo sapiens* [GenBank:NC_000018.9], *Bos taurus* [GenBank:GJ062838.1], *Arabidopsis thaliana* GenBank:NC_003071.7], *Daucus carota* [GenBank:AJ003139.1]. Click here for file
