# Supplementary material for: Trichinella pseudospiralis vs. T. spiralis thymidylate synthase gene structure and T. pseudospiralis thymidylate synthase retrogene sequence
Source: Parasit Vectors. 2014 Apr 9;7:175. doi: 10.1186/1756-3305-7-175 (PMC4022200; doi:10.1186/1756-3305-7-175)
Supplement: Additional file 1: Figure S1 — Alignment of T. spiralis and T. pseudospiralis thymidylate synthases exon (bold) and intron (italic) sequences. Additionally, a 98 nt-long fragment of the T. spiralis gene 5′ flanking region is shown, with putative TATA box underlined, and 29 nt-long fragment, corresponding to 3′ UTR [16], is shown as the gene 3′ flanking region. The whole T. spiralis and T. pseudospiralis gene sequences are available through the accessions [GenBank:AF406808] and [GenBank:KF186231], respectively. Nucleotide positions differing between the genes of two species are indicated by asterisks. Alignment was performed using Clustal X software and Genomatix MatInspector software served for consensus TATA box identification. Figure S2. Alignment of T. spiralis and T. pseudospiralis thymidylate synthase amino acid sequences. Amino acid substitutions are marked with asterisks. Enzyme conserved folate and doexyuridylate (active center) binding sites are marked bold. Figure S3. Alignment of amino acid sequences of various thymidylate synthases performed in Clustal X software. The intron positions are marked as boxes for phase 0 introns, elipses for phase 1 introns or pentagons for phase 2 introns. The sequences were obtained via the following accessions: Trichinella spiralis [GenBank:AF406808.3], Trichinella pseudospiralis [GenBank:KF186231], Brugia malayi [GenBank:NW_001893010], Loa loa [Genbank:NW_003320690], Caenorhabditis elegans [GenBank:AF099673.1], Filobasidiella neoformans [GenBank:U12256.1], Pneumocystis carinii [Genbank:M25415.1], Rattus norvegicus [GenBank:NC_005108.3], Mus musculus [GenBank:NW_001030787.1], Homo sapiens [GenBank:NC_000018.9], Bos taurus [GenBank:GJ062838.1], Arabidopsis thaliana GenBank:NC_003071.7], Daucus carota [GenBank:AJ003139.1]. [file 1756-3305-7-175-S1.doc]

Supplementary Figure S1. Alignment of *T. spiralis* and *T. pseudospiralis* thymidylate synthases exon (bold) and intron (italic) sequences. Additionally, a 98 nt-long fragment of the *T. spiralis* gene 5’ flanking region is shown, with putative TATA box underlined, and 29 nt-long fragment, corresponding to 3’ UTR [16], is shown as the gene 3’ flanking region. The whole *T. spiralis* and *T. pseudospiralis* gene sequences are available through the accessions [GenBank:AF406808] and [GenBank:KF186231], respectively. Nucleotide positions differing between the genes of two species are indicated by asterisks. Alignment was performed using Clustal X software and Genomatix MatInspector software served for consensus TATA box identification.

T.sp. -98 *AACGTATATACGATTGATTATAGTCGACGATTGTTCGACCCTAGTTCTTCTAGTTGTCTTACAGTATTTGCATACTTGAATTTATAGATATTTATACA* 0

T.psp. --------------------------------------------------------------------------------------------------

+1

T.sp**.** 1 **ATG ACA GAA ACT GTT CAC AAA TTA GAT ACT AAT TCA ACA TCT CAG GAT GAT TAC GTG AA** *GTTCGTTTATTAATTCATT* 78

T.psp. **ATG ACA GAA ACT GTT** **CAC AAA TTA GAT AGT AAT TCA ACA TCT CAG CAT GAT TAC GTG AA** *GTTAGTG-AAAAATTTATT*

* *

T.sp. 79 *CAACATTATGAAGTTATTTTGTTAACTGTAAACATATTTAG* **T CAG GAG GAA CTG AAT TAT TTG** **AAT CAA CTG AAG GAC ATA ATT** 162

T.psp. *CAACATTATGAAGTTATTTTGTTGAATATAAATATATAAAG* **T CAG GAA GAA TTG AAT CAT TTG** **AAT CAA CTG AAG GAC ATA ATT**

* * *

T.sp. 163 **GAT CAC GGT GTT CGT AAA AAC GAT CGT ACT GGA ATC GGA ACA TTG TCA** **ACT** **TTT GGA ACG CAG TCT CGG TAT TGC** 237

T.psp. **GAT CAT GGC GTC CGT AAA AAC GAT CGT ACT GGG ATT GGA ACG TTG TCA** **ACT TTT** **GGA ACG CAA TCC CGC TAT TGC**

* * * * * * * * *

T.sp. 238 **CTT CGA GAT** **G** *GTAGCAGATTTTTAAACTGAAATTTAATTTCAGTCGATGAGAAATGTTATTTGCAG* **AT ATT** **TTT CCG** **CTT CTT ACA A** 324

T.psp. **CTT CGA GAT G** *GTAGCAAATTTTTAAAATGAAATTTAACTTTTATCAATAAGAAAT--TGTTTGAAG* **AT ATT** **TTT CCA** **CTG CTT ACA A**

* *

T.sp. 325 **CA AAA CGC GTC TTT TGG AGA GGA GTG GTC GAA GAA CTG CTT TGG TTC ATC AGT GGA AGC ACC AAT GCT AAA CAG** 398

T.psp. **CA AAA CGC GTC TTT TGG AGA GGA GTG GTT GAA GAA TTG CTT TGG TTC ATC AGT GGA AAC ACG AAT GCG AAA AAG**

* * * * * *

T.sp 399 **CTT TCA GAG AAG AAT GTC AAC ATT TGG GAT GGA AAT TCT TCC AGA GAA TTC CTA GAC AGT AGA GGA CTG TAC AAT** 473

T.psp. **CTT TCA** **GAG AAG AAT GTT AAC ATC TGG GAT GGA AAT TCT TCC AGA GAA TTC CTG GAC AGT AGA GGG CTG TAT AAT**

* * * * *

T.sp. 474 **TAT GAA GAA GGT GAT TTG GGG** **CCT** **GTT TAT GGT TTC CAA TGG CGC CAC TTT GGA TGC CCG TAT TCA TCG ATG ACG** 548

T.psp. **TAT GAA GAA GGT GAC TTG GGG** **CCT GTT TAT GGC TTT CAA TGG CGC CAC TTT GGG TAT CCG TAT ACA TCT ATG ACG**

* * * * ** * *

T.sp. 549 **GCC GAT TAT AAA GGA AAA GGC TAC GAT CAA TTA CAA** **CAG** **TGT ATT AAA ATG ATT CGT GAA GAA CCG GAA AGT CGA** 623

T.psp. **GCC GAT TAT GCA GGA AAA GGC TAT GAT CAA TTA CAA** **CAG TGT ATT AAA ATG ATT CGT GAA GAA CCG GAA AGT CGA**

** *

T.sp. 624 **CGA ATC ATC ATG ACC GCA TGG AAT CCC TGT G** *GTTAGTTTGATGGAAAATTTATGTATGCATTCTGTTGATTTCTCTTTTTCAG* **AT T** 709

T.psp. **CGA ATC ATC ATG ACT GCA TGG AAT CCG TGT G** *GTTAGTGTGATGGAAAATTTATGTATGCATTCTGTTGATTTGTTATTTTCAG* **AT T**

* *

T.sp. 710 **TA GAG AAA GTC GCT TTG CCA CCT TGT CAC TGT TTC GTC CAA TTT TAC GTT GCT GAT GGG GAG CTA TCT** **TGT CAA** 783

T.psp. **TA GAG AAA GTG GCT TTG CCA CCT TGT CAC TGT TTC GTC CAA TTT TAC GTT GCT GAT GGG GAA CTA TCT** **TGT CAA**

* *

T.sp. 784 **ATG TAT CAA CGA AGT GCA GAT ATG** *GTATTCTTCTTTTCACATGTTTAAACTTGTTTACCTTGTAAAATAATATTCAG* **GGA TTG GGA G** 870

T.psp. **ATG TAT CAA CGA AGT GCA GAT ATG** *GTATTCTTCTTTTCATATTTTTAAACTTGTTTACCTTGTAAAA-AATATTCAG* **GGT TTG GGA G**

*

T.sp. 871 **TG CCG TTT** **AAC ATT GCC AGC TAT TCT TTA CTG ACA CGT ATG ATA GCC CAC ATT ACT TCA CTG AAG CCA GGG TTT** 944

T.psp. **TG CCG TTT** **AAC ATT GCC AGC TAT TCT TTA CTG ACA CGT ATG ATA GCC CAT ATT ACT TCA TTG AAA CCT GGT TTT**

* * * * *

T.sp. 945 **TTC ATA CAT ACA ATC GGA GAT GCG** **CAT GTC TAC TTA ACA CAT GTT GAC GCT TTG AAA GTT CAA** *GTAAAAATGAAAATA* 1022

T.psp. **TTC ATA CAT ACA ATC GGA GAT GCG** **CAT ATC TAC TTA ACA CAT GTC GAC GCT TTG AAA GTT CAA** *GTAAAA-------TA*

* *

T.sp. 1023 *ATATGCTTATTTTTAAACTTTTCGAAAGTATGTAAAATTATTTA–ATTCATTAATGTCTGTTTA------------G* **ATG** **GAA AGA AAA CCA A** 1102

T.psp. *AAATACTT------AAAATTTTTAAAAGTATTTAAAATTATTTATATTCATGAATGTCTGTTTATTTATTATTATAG* **TTG GAG AGA AAA CCA A**

* *

T.sp. 1103 **GA CCG TTT CCG AAG CTG AAA ATT TTA AGA AAT GTG GAA AAT ATT GAT GAT TTT CGG** **GCT GAA GAC TTT GAA TTA** 1176

T.psp. **GA CCG TTT CCG AAG CTG AAA ATT TTA AGA AAT GTG GAA AAT ATT GAT GAT TTT AGG** **GCT GAA GAC TTT GAA TTA**

*

T.sp. 1177 **ATT AAT TAT AAA CCA TAT CCA AAA ATA TCA ATG CCT ATG GCT GTG TGA** *TCAATTGAAAAGAAATAAAATTTCATGGT* 1253

T.psp. **ATT GAT TAT AAA CCA TAT CCA AAA ATA TCA ATG CCT ATG GCT GTG TGA** -----------------------------

*

Supplementary Figure S2. Alignment of *T. spiralis* and *T. pseudospiralis* thymidylate synthase amino acid sequences. Amino acid substitutions are marked with asterisks. Enzyme conserved folate and doexyuridylate (active center), binding sites are marked bold.


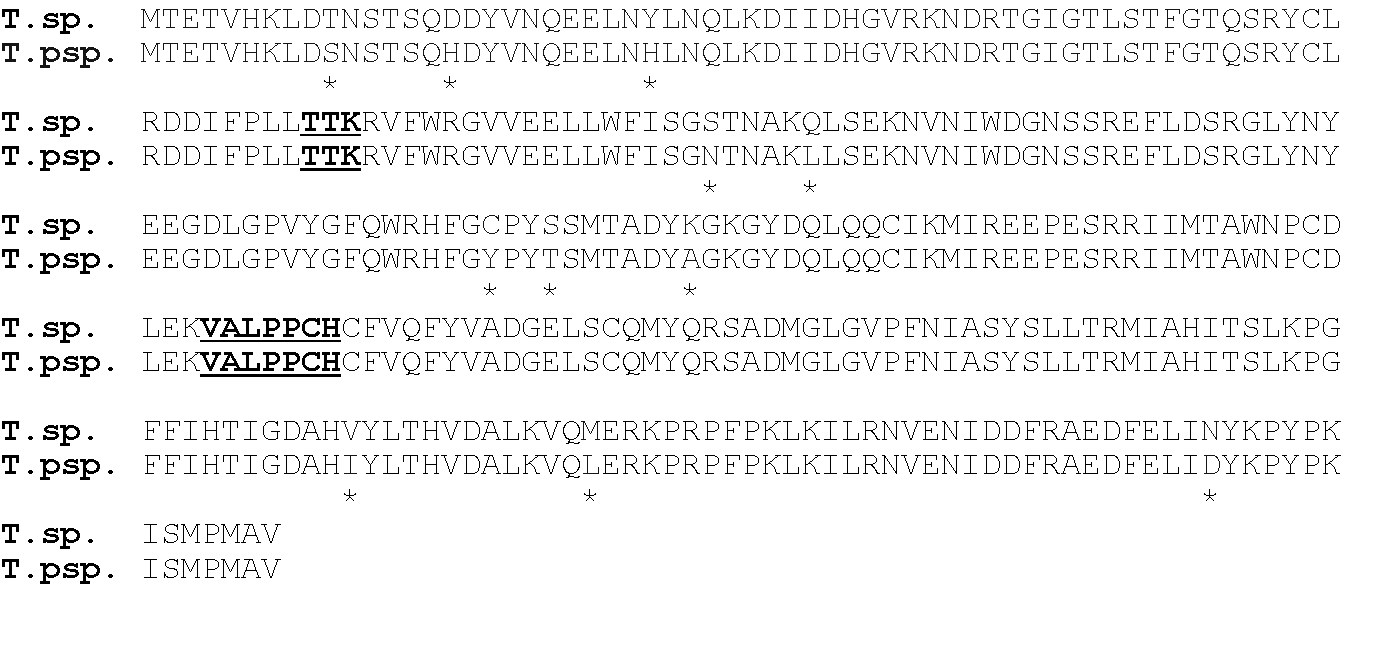


Supplementary Figure S3. Alignment of amino acid sequences of various thymidylate synthases, performed in Clustal X software. The intron positions are marked as boxes for phase 0 introns, elipses for phase 1 introns or pentagons for phase 2 introns. The sequences were obtained via the following accessions: *Trichinella spiralis* [GenBank:AF406808.3]*, Trichinella pseudospiralis* [GenBank:KF186231], *Brugia malayi* [GenBank:NW_001893010], *Loa loa* [Genbank:NW_003320690], *Caenorhabditis elegans* [GenBank:AF099673.1], *Filobasidiella neoformans* [GenBank:U12256.1], *Pneumocystis carinii* [Genbank:M25415.1], *Rattus norvegicus* [GenBank:NC_005108.3], *Mus musculus* [GenBank:NW_001030787.1], *Homo sapiens* [GenBank:NC_000018.9], *Bos taurus* [GenBank:GJ062838.1], *Arabidopsis thaliana* GenBank:NC_003071.7], *Daucus carota* [GenBank:AJ003139.1].

M. musculus ------------------------------------------------------------

R. norvegicus ------------------------------------------------------------

H. sapiens ------------------------------------------------------------

B. taurus ------------------------------------------------------------

C. elegans ------------------------------------------------------------

A. thaliana -----------------------------MATTTLNDS-------VTTTLASE-------

D. carota MYKALISSTFPLLNSLSQTLALRSASLYLLTNCNLSSSNFSHCHRFTTYMASERLANPTN

**T. spiralis ------------------------------------------------------------**

L. loa ------------------------------------------------------------

B. malayi ------------------------------------------------------------

F. neoformans ------------------------------------------------------------

P. carinii ------------------------------------------------------------

M. musculus ------------------------------------------------------------

R. norvegicus ------------------------------------------------------------

H. sapiens ------------------------------------------------------------

B. taurus ------------------------------------------------------------

C. elegans ------------------------------------------------------------

A. thaliana --------PQRTYQVVVAATKEMGIGKDGKLPWNLPTDLKFFKDITLTTSDSSKKNAVVM

D. carota GSGITRPDPQRTYQVVVAATQNMGIGKDGKLPWRLPSDMKFFKDVTMTTSDPLKRNAVIM

**T. spiralis ------------------------------------------------------------**

L. loa ------------------------------------------------------------

B. malayi ------------------------------------------------------------

F. neoformans ------------------------------------------------------------

P. carinii ------------------------------------------------------------

M. musculus ------------------------------------------------------------

R. norvegicus ------------------------------------------------------------

H. sapiens ------------------------------------------------------------

B. taurus ------------------------------------------------------------

C. elegans ------------------------------------------------------------

A. thaliana GRKTWESIPIKYRPLSGRLNVVLTRSGGFDIANTENVVTCSSVDSALDLLRAPPYCLSIE

D. carota GRKTWESIPIQHRPLPGRLNVVLTRSGSFDIATVENVVICGSMISALELLAGSPYCVSVE

**T. spiralis ------------------------------------------------------------**

L. loa ------------------------------------------------------------

B. malayi ------------------------------------------------------------

F. neoformans ------------------------------------------------------------

P. carinii ------------------------------------------------------------

M. musculus ----------------------------------------------------MLVVGSE-

R. norvegicus ----------------------------------------------------MLVEGSE-

H. sapiens ----------------------------------------------------MPVAGSEL

B. taurus ----------------------------------------------------MPAAGSEP

C. elegans ----------------------------------------------------MEVMNKEN

A. thaliana RVFVIGGGDILREALNRPSCDAIHLTEIDTSVDCDTFIPAIDTSVYQPWSSSFPVTENGL

D. carota KVFVIGGGQIYREALNAPGCDAVHITEIEEHIECDTFIPLLDESVFQPWYSSFPLVENKI

**T. spiralis ------------------------------------------------------------**

L. loa ------------------------------------------------------------

B. malayi ------------------------------------------------------------

F. neoformans ------------------------------------------------------------

P. carinii ------------------------------------------------------------

M. musculus ---------LQSDAQQLSAEA--------------------------PRHGELQYLRQVE

R. norvegicus ---------LQSGAQQPRTEA--------------------------PQHGELQYLRQVE

H. sapiens -----PRRPLPPAAQERDAEPR-------------------------PPHGELQYLGQIQ

B. taurus -----SRPPSPPGVQEQSAEPRPPPP---------------------PPHGELQYLGQIE

C. elegans --------IIADAPSDVVKTVQQQV-------------------VH-LNQDEYKYLKQVE

A. thaliana RFCFTTFVRVKSSADESSDESNGSQS-----LQFDGKKFLFLPKMVFDQHEEFLYLNMVE

D. carota RYCFTTYVRVRNSVAELTSQTNGCSSDSKSDSNFEIQNFSFLPKTVFLPKTVFEKHEEYL

**T. spiralis ---------MTETVHKLDTNSTSQDD--------------------YVNQEELNYLNQLK**

L. loa ---------MKSL--KDNGNDGDTDVLV--------------------NEDENKYLDQIR

B. malayi ---------MKSS-GAHGNVMGDAGVLK--------------------NEDESKYLDQVR

F. neoformans ---------MTATIDDQEKNQ-----------------------RSNPDHEEYQYLDLIR

P. carinii ----------------------------------------------MVNAEEQQYLNLVQ

M. musculus HILRCGFKKEDRTGTGTLSVFG-MQARYSLRD-EFPLLTTKRVFWKGVLEELLWFIKGST

R. norvegicus HIMRCGFKKEDRTGTGTLSVFG-MQARYSLRD-EFPLLTTKRVFWKGVLEELLWFIKGST

H. sapiens HILRCGVRKDDRTGTGTLSVFG-MQARYSLRD-EFPLLTTKRVFWKGVLEELLWFIKGST

B. taurus HILRCGFRRDDRTGTGTLSVFG-MQARYNLRD-EFPLLTTKRVFWKGVLEELLWFIKGST

C. elegans QILREGTRRDDRTGTGTISIFG-MQSKYCLRNGTIPLLTTKRVYWKGVLEELLWFISGST

A. thaliana DIISNGNVKNDRTGTGTLSKFG-CQMKFNLRRS-FPLLTTKRVFWRGVVEELLWFISGST

D. carota NIISNGVTKNDRTRTGTVSIFG-CQMRFNLRKS-FPLLTTKKVFWRGVVEELLWFISGST

**T. spiralis DIIDHGVRKNDRTGIGTLSTFG-TQSRYCLRDDIFPLLTTKRVFWRGVVEELLWFISGST**

L. loa HILKNGERIDDRTGVGTISVFG-MHSVYSLRNGVVPVLTTKRVYWKGVVEELLWFIRGDT

B. malayi YILKNGERIDDRTGVGTISVFG-MHSVYSLRNGVVPVLTTKRVYWKGVVEELLWFIRGDT

F. neoformans RIINVGEVRPDRTGTGTVALFAPPSFRFSLADNTLPLLTTKRVFLRGVIAELLWFVSGCT

P. carinii YIINHGEDRPDRTGTGTLSVFAPSPLKFSLRNKTFPLLTTKRVFIRGVIEELLWFIRGET

M. musculus NAKELSSKGVRIWDANGSRDFLDSLGFSARQEGDLGPVYGFQWRHFGAEYKDMDSDYSGQ

R. norvegicus NAKELSSKGVRIWDANGSRDFLDSLGFSARQEGDLGPVYGFQWRHFGADYKDMDSDYSGQ

H. sapiens NAKELSSKGVKIWDANGSRDFLDSLGFSTREEGDLGPVYGFQWRHFGAEYRDMESDYSGQ

B. taurus NAKRLSSKGVKIWDANGSRDFLDGLGFSDRAEGDLGPVYGFQWRHFGAEYKDMDSEYSGQ

C. elegans DGKLLMEKNVKIWEKNGDRAFLDNLGFTSREEGDLGPVYGFQWRHFGAKYVDCHTDYSGQ

A. thaliana NAKVLQEKGIHIWDGNASREYLDGIGLTEREEGDLGPVYGFQWRHFGAKYTDMHADYTGQ

D. carota NAKILKEKGVNIWEGNGSREYLDSIGLTDREEGDLGPIYGFQWRHFGARYTDMHADYSGQ

**T. spiralis NAKQLSEKNVNIWDGNSSREFLDSRGLYNYEEGDLGPVYGFQWRHFGCPYSSMTADYKGK**

L. loa NAKHLSEKGVRIWDANGSRQFLDQCGFNDRSEGDLGPVYGFQWRHCGAEYRGMDADYTNR

B. malayi NAKHLSEKGVRIWDANGSRQFLDQCGFSDRSEGDLGPIYGFQWRHCGAEYRGMDTDYTNQ

F. neoformans DAKMLSSQGVGIWDGNGSKEFLEKVGLGHRREGDLGPVYGFQWRHFGAEYTDADGDYKGK

P. carinii DSLKLREKNIHIWDANGSREYLDSIGLTKRQEGDLGPIYGFQWRHFGAEYIDCKTNYIGQ

M. musculus GVDQLQKVIDTIKTNPDDRRIIMCAWNPKDLPLMALPPCHALCQFYVVN-----------

R. norvegicus GVDQLQKVIDTIKTNPDDRRIIMCAWNPKDLPLMALPPCHALCQFYVVN-----------

H. sapiens GVDQLQRVIDTIKTNPDDRRIIMCAWNPRDLPLMALPPCHALCQFYVVN-----------

B. taurus GVDQLQKVIDTIKTNPNDRRILLCAWNPKDLPLMALPPCHALCQFYVVN-----------

C. elegans GVDQLAEVIRQIKEQPDSRRIIMSAWNPSDLGQMVLPPCHTMCQFYVDN-----------

A. thaliana GFDQLVDVIDKIKNNPDDRRIIMSAWNPSDLKLMALPPCHMFAQFYVAE-----------

D. carota GFDQLLDVISKIKNNPDDRRIIQSAWNPSDLRLMALPPCHMFAQFYVAN-----------

**T. spiralis GYDQLQQCIKMIREEPESRRIIMTAWNPCDLEKVALPPCHCFVQFYVAD-----------**

L. loa GIDQLSEIIDLIKNEPHSRRIILSAWNVKDLKLMVLPPCHTLAQFAVRN-----------

B. malayi GIDQLSEIIDLIKNEPHSRRIILSAWNVKDLKLMALPPCHTLAQFAVRN-----------

F. neoformans GVDQLQRVIDTIKNNPTDRRIILSAWNPKDLPLMALPPCHMFCQFFVSLPP--------A

P. carinii GVDQLANIIQKIRTSPYDRRLILSAWNPADLEKMALPPCHMFCQFYVHIPS---------

M. musculus ------GELSCQLYQRSGDMGLGVPFNIASYALLTYMIAHITGLQPGDFVHTLGDAHIYL

R. norvegicus ------GELSCQLYQRSGDMGLGVPFNIASYALLTYMIAHITGLQPGDFVHTLGDAHIYL

H. sapiens ------SELSCQLYQRSGDMGLGVPFNIASYALLTYMIAHITGLKPGDFIHTLGDAHIYL

B. taurus ------GELSCQLYQRSGDMGLGVPFNIASYALLTYMIAHITDLKPGDFVHTLGDAHIYL

C. elegans ------GELSCQLYQRSGDMGLGVPFNLASYGLLTHMIAKVCGLKPGTLVHTLGDAHVYS

A. thaliana ------GELSCQMYQRSADMGLGVPFNIASYSLLTCMLAHVCDLVPGDFIHVLGDAHVYK

D. carota ------GELSCQMYQRSADMGLGVPFNIAAYALLTCMIAHVCDLVPGDFVHSIGDAHVYS

**T. spiralis ------GELSCQMYQRSADMGLGVPFNIASYSLLTRMIAHITSLKPGFFIHTIGDAHVYL**

L. loa ------DELSCQLYQRSGDMGLGVPFNLASYGLLTHMIAHICGLKAGYLCHVLGDAHVYV

B. malayi ------GELSCQLYQRSGDMGLGVPFNLASYGLLTHMIAHVCGLKTGHLCHVLGDAHVYM

F. neoformans DSPGSKPKLSCLMYQRSCDLGLGVPFNIASYALLTHMIALITDTEPHEFILQMGDAHVYR

P. carinii N--NHRPELSCQLYQRSCDMGLGVPFNIASYALLTCMIAHVCDLDPGDFIHVMGDCHIYK

M. musculus NHIEPLKIQ-------------------------------------LQREPRPFPKLKILR

R. norvegicus NHIEPLKIQ-------------------------------------LQREPRPFPKLRILR

H. sapiens NHIEPLKIQ-------------------------------------LQREPRPFPKLRILR

B. taurus NHIEPLKTQALMELRGQSSRSLDGDQAGTSRWAPVATDTERDRCCELQREPRPFPKLKILR

C. elegans NHVDALKIQ-------------------------------------LDREPYAFPKIRFTR

A. thaliana THVRPLQEQ-------------------------------------LLNPPKPFPVLKINP

D. carota NHLSDLFET------------------------------------SFRMLPKTFPVLKINS

**T. spiralis THVDALKVQ-------------------------------------MERKPRPFPKLKILR**

L. loa NHVDALQEQ-------------------------------------LRRQPRTFPTVRFVG

B. malayi NHVDALQEQ-------------------------------------KKRQPRQFPTVRFIG

F. neoformans DHVEPLKTQ-------------------------------------LEREPRDFPKLKWAR

P. carinii DHIEALQQQ-------------------------------------LTRSPRPFPTLSLNR

M. musculus K---VETIDDFKVEDFQIEGYNPHPTIKMEMAV

R. norvegicus K---VETIDDFKVEDFQIEGYNPHPTIKMEMAV

H. sapiens K---VEKIDDFKAEDFQIEGYNPHPTIKMEMAV

B. taurus K---VETIDDFQAEDFQIEGYNPHPTIKMEMAV

C. elegans D---VASIDDFTSDMIALDDYKCHPKIPMDMAV

A. thaliana E---KKQIDSFVASDFDLTGYDPHKKIEMKMAV

D. carota G---EKDIDSFEAADFKLIGYDPHQKIEMKMAV

**T. spiralis N---VENIDDFRAEDFELINYKPYPKISMPMAV**

L. loa N---IKTIDDFTYESIVLENYQPMPAIKMAMA-

B. malayi N---IKTIDDFTSDMIALDDYKCHPKIPMDMAV

F. neoformans SKEEIGDIDGFKVEDFVVEGYKPWGKIDMKMSA

P. carinii S---ITDIEDFTLDDFNIQNYHPYETIKMKMSI
